# Brief communication: qualitative evaluation of call-for-life mHealth tool among youth living with HIV in Uganda

**DOI:** 10.1186/s12981-025-00798-6

**Published:** 2025-10-21

**Authors:** Agnes Bwanika Naggirinya, Joseph Rujumba, Joshua Beinomugisha, Suzan Nakazzi, Peter Waiswa, David B. Meya, Rosalind Parkes-Ratanshi

**Affiliations:** 1 Department of Medicine, College of Health Sciences, Makerere University, Hall Lane, P.O.Box 22418, Kampala, Uganda; 2 Infectious Diseases Institute, Academy for Health Innovations, Makerere University, Kampala, Uganda; 3 Department of Pediatrics and Child Health, College of Health Sciences, Makerere University, Kampala, Uganda; 4 Department of Health Policy, Planning, and Management, School of Public Health, College of Health Sciences, Makerere University, Kampala, Uganda; 5 Department of Global Health, Queen’s University, Belfast, UK

**Keywords:** Youth, HIV, Adherence, Motivation, Behavioural skills, Mhealth, Call for life, IVR

## Abstract

**Introduction:**

Afew studies have assessed the acceptability of mHealth interventions in youth living with HIV, Call For Life -Interactive Voice Response (C4L-IVR) system developed to support patients with HIV and TB in Uganda, specifically to improve treatment adherence and retention in care. This qualitative study examined the acceptability and usage of C4L-IVR, barriers and enablers of adherence and retention in care among youth living with HIV in rural Uganda.

**Methods:**

Nested within a randomised intervention trial (NCT 04718974), this qualitative study examined youth 16–24 years old, through focus group discussions and indepth interviews at study end. Induction and deduction analysis was done with support of Nvivo software guided by the information motivation behavioral theory.

**Results:**

Between 9th December 2021 and 28th Apr 2022, 68 participants were recruited, with 38 (56%) females. We conducted 14 interviews 7 focused group discussions (FGD) (02 female only FGD; 5 mixed); and 7 in-depth interviews (IDI) (4 males and 3 females IDI. Seven main themes were identified: information received from the system, motivation from system calls, behavioural skills, barriers to adherence and retention, acceptability of the tool, experiences, and suggestions for improvement. All youth accepted the tool, scoring it highly comfortable on a 5 Likert scale, where 5 was “very comfortable” and 1 “not at all comfortable'.

**Conclusion:**

The high acceptability and usage of C4L system along with impact on behavioral skills, this system had more enablers than barriers to ART adherence and retention in care.

**Supplementary Information:**

The online version contains supplementary material available at 10.1186/s12981-025-00798-6.

## Main text

### Introduction

World Health Organization (WHO) has endorsed text messaging or short message service (SMS) interventions for supporting individual-level adherence to ART and to improve linkage of people diagnosed with HIV to HIV-care services [23]. Previous mHealth and Call for life studies have shown improved virological outcomes [3], reduced non-adherence risk, improved quality of life [5] through ART adherence and clinic reminder support, and increased self-reported adherence among non-adherent youth [9] 

However, few studies have assessed the acceptability of mHealth intervention in young people living with HIV [15], the studies were mainly in urban settings and very few in rural areas. Whereas most studies found a high acceptability, there are still factors influencing acceptability and these include ehealth literacy and readiness [20]; technology-associated barriers, privacy, safety and security [2, 11]. The factors influencing acceptability also impact on the utilization of the mHealth intervention, especially the frequency and effective use of this intervention [24].

Following the completion of a randomized clinical study (NCT 04718974) [4], we looked at the acceptability and usage of Call For Life -Interactive Voice Response (C4L-IVR) among young men and women living with HIV in rural Uganda randomized to C4L-IVR arm and the barriers and enablers they face while using the system. We obtained the usage data from the system over the past 12 months.

The qualitative interviews guided by information, motivation, and behavioral skills model, were analysed with support of NVivo software and overarcing themes on acceptability, enablers or barriers to using C4L-IVR for adherence and retention in care are reported..

### Methods

This was a cross-sectional qualitative study nested within a randomized clinical trial (NCT 04718974), and it was conducted in Kiryandongo district, where three study sites were purposively chosen to represent different levels of health facilities involved in HIV care in the district, namely; Kiryandongo Hospital, Panyadoli Health Centre III, and Nyakadoti Health Centre II. The intervention in this study was Call for Life Interactive Voice Response Tool, a software that is based on open-source Mobile Technology for Community Health (MoTeCH). The Call for life-Interactive Voice Response (C4L-IVR) system, an automated phone-based technology, used a basic analog phone, sent out pre-recorded messages with voice prompts, allowed participants to provide feedback using their phone keypads, ably scheduled calls at predetermined times and route calls through any phone network.

The study enrolleded young adults 15–24-year-olds living with HIV. The participants were part of a randomized clinical trial of an interactive voice response tool registered on the intervention at any of the three study sites [4].

Data from the system for the past study period was downloaded and usage/interaction with the system was grouped based on participant’s engagement with the system, data collection was through focus group discussions and in-depth interviews. Interviewers included a Ph.D student, a medical doctor, a social scientist, and a male qualitative researcher. The aim was to understand participants' engagement with the C4L-IVR intervention, its impact on adherence, retention behaviors, frequency and adequacy of use, and acceptance or resistance.

Participants were categorized based on usage and engagement with the C4L-IVR tool, with 50% engagement and viral load outcome at month 12. Six focus group discussions with 7–10 participants and five in-depth interviews were conducted, all interviews were recorded with permission, FGD lasted on average one hour forty five minutes and IDI forty minutes on average.

Data analysis was guided by the information motivation behavior skills model (IMB) [8]. The IMB model was used to understand the social and psychological factors that promoted health-related behavior to improve ART adherence and retention in care among youth on the intervention.

The audio scripts were transcribed, imported and analyzed using deductive and inductive thematic framework analysis with support of NVivo 14.0 software. Throughout the process of data handling and interpretation, the identities of qualitative participants were masked to maintain anonymity.We report the overarching themes, subthemes and recommendations.

### Results

Between December 2021 and April 2022, 65 participants were selected for endline qualitative interviews. The majority were females, aged 19–25, with a steady sexual partner, and 60% having primary education. The interviews included 7 focused group discussions and 7 in-depth interviews (Table 1**).**

**Table 1:** Socio-demographic characteristics of participants

Group category	Numer of participants(%)
Age	
19–21	13(20%)
22–25	52(80%)
Gender	
Female	49(75%)
Male	16(25%)
Marital status	
Steady partner	46(71%)
Separated/divorced/widowed	04(6%)
Single	15(23%)
Education Status	
Primary Level	39(60%)
Secondary Level	23(35%)
Tertiary level	03(5%)
Occupation	
Yes	55(85%)
No	10(15%)

#### Major themes as guided by the IMB model

The study identified seven main themes: information received from the system, motivation from system calls, behavioral skills, barriers to adherence and retention, acceptability of the tool, experiences while on the system, and suggestions for tool improvement. The major theme was motivation received from the Call for Life system, followed by information, acceptability of the system, impacted behaviors, experiences while on the tool, barriers to adherence faced by the youth, and suggestions for improvement.

##### Acceptability

This was assessed based on downloaded system data on percentage engagement with the system, and reported comfort. The comfort was assessed using a Likert scale of 1–5 with 5 “very comfortable”; 4 “ comfortable”, 3 “neutral- neither comfortable nor uncomfortable”, “ 2 “somewhat comfortable” and 1 “not at all comfortable” was used to assess comfort and ease of use. Acceptability based on a percentage of engagement for the scheduled calls- was an aggregate for each functionality- health tips calls, adherence calls and appointment reminder calls from downloaded system data,

Four subthemes emerged under the acceptability theme: ease of use, usefulness, engagement, and admissible options. All youth accepted the call for life interactive voice response system (C4L-IVR) and scored the comfort of use on a likert scale of 1–5, where 5 was “very comfortable”; 4 “ comfortable”, 3 “neutral- neither comfortable nor uncomfortable”, “ 2 “somewhat comfortable” and 1 “not at all comfortable”, with some giving a percentage mark. The youth's opinions on the tool were based on likert scores.

“*So this system has been easy and is very simple and is easy to understand. Even a person who is a slow learner will understand ……….So it is very easy and very simple*” (Youth day 2 afternoon FGD)

“*If it was giving percentages I could give 90% because this C4L has helped me a lot, not only on the time for taking medicine but it has been giving me health tips …… I had a lot of fears*” (Youth Day 3 afternoon FGD)

##### Barriers and enablers to adherence and retention

This describes the barriers to ART adherence and retention in care despite participants being registered on the IVR system.

The subthemes to barriers to adherence and retention in care included; travels away from home, fear of pills, fear of unintended disclosure when seen swallowing pills, and stigma associated with attending clinics.

*“The youths, are scared, sometimes think that if they go to a health facility, they will meet a friend who will disclose their status. They fear to line up for drugs because of unintended disclosure. But if put on C4L, they will learn from the teachings and become strong and courageous and be able to pick drugs”* ( Youth Day 3 afternoon FGD)

##### Behavioral skills

This describes the Behavioral skills gained from the IVR system to perform the behaviours of pill adherence, appointment keeping, and positive living.

On behavioral skills gained from being on the intervention, the subthemes included: use of condoms, reduced sexual partners, eating a balanced diet to counter the effects of ART, conditioned to swallow the pills as soon as the call comes in, and always taking medicines as per prescription.

*“They told me if you are pregnant, you are supposed to take medicine on time and also am not supposed to have many sexual partners, that’s what I am doing”* (IDI at study end)

##### Information theme

Describes the information and knowledge received to impact behavioural change.

For information received from the health infotips, the subthemes included advice on prevention of mother-to-child transmission (PMTCT), breastfeeding when HIV positive, nutrition in HIV, TB screening, abstinence, and positive living.

*“ I've learned how to take medications properly and on time. I've also learned how to breastfeed my baby, so he doesn't get HIV. I've learned how to protect my husband. In the event that I am diagnosed with tuberculosis, I have to take my medication as directed.”* (Youth FGD, Day 1 Morning)

##### Motivation

This theme describes motivation got from IVR system to perform the behaviours of picking medications, clinic visits and swallowing pills on time.

Under thistheme, three subthemes emerged, these included the remote psychosocial support from voice calls, remote symptom reporting with feedback from project staff, and the ability to share reminder calls and health infotips for those in concordant relationships.

*“Because of the advice given to us through the phone, I started accepting my situation and living positively, I am no longer afraid. When the time for picking up drugs is due, one week to the appointment date, they call to remind me.”* (Youth FGD-Day 2 afternoon)

#### Challenges faced while using the IVR system and suggestions for improvement 

Challenges on the system, were mainly phone-based challenges, and these included maneuvering at night during the dark to locate the dial pad, which often resulted in wrong response options, being locked out on the third attempt, lack of electricity forced them to incur costs for battery charging and afew reported limited language on the system.

*“I didn’t know how to use the phone very well and the phone buttons were hard*”( FGD Day 3 morning)

*“What I can say bad about the call for lifel, it rings like several times and you have to input the 4digits and if you forget one number, it ends and rings again and when you put a password it tells you that it is locked; that’s what I see is bad about it” (*FGD Day 1 morning*)*

*“Sometimes they can fear to take medicine because they did not have advice that this system is giving. But if they register them to that system, it will help them to remind them and to give them advise”(*FGD Day 2 afternoon*)*

### Discussion

The "Call for life" mHealth tool use among young people living with HIV was found to be permissible, acceptable, and easy to use,this has been documented elsewhere in both developed [15] [1, 19, 22] and developing countries [26] [7, 17]. The youth embraced the daily pill reminder function, which helped reduce forgetfulness, a barrier to pill adherence. The C4L-IVR system also impacted young adults' health behaviors, particularly in sexual behavior, nutrition habits, attitude towards prescribed medication, and adherence to pills. However, there is no documented mHealth study that has reported on safer sex behavior change or nutrition habits among young people living with HIV and afew on adherence behaviors [21].

The study used the IMB behavioral skills model to analyze motivational support for HIV care among youth. Voice health tips provided psychosocial support on positive living, remote symptom reporting, and management. Pill reminders encouraged discordant relationships and nursing mothers to protect loved ones. A similar design in Rhode Island involved iPhone gaming intervention, providing comprehensive information about HIV, clinic visits, and general health information. However, barriers to ART included fear of pills, unplanned travels, unintended disclosure, sideeffects and stigma. These barriers have been reported in previous studies, including those in Zambia, India, the USA and Ethiopia [6, 10, 13, 18].

Despite a high penetration of mobile phones[14], a good number of young people in rural areas possess no personal phones; to overcome bias, every young person in the study was given a feature mobile phone. In this study, the young people reported various experiences and the negative experiences were reported by the first-time mobile phone users, some of these challenges were due to infrastructure like lack of electricity which led to costs on charging the phone battery [25], the lack of electricity has been reported in other studies in Uganda [12, 25], entry of wrong pin codes especially in the night and poor network [16]which often led to repeated outbound calls.

The study aimed to understand the experiences of both new and established youth on ART and the factors that support and hinder adherence to the Call for Life tool. It also highlighted the importance of low-level medical facilities in providing healthcare. However, the study's limitations include a small sample size, non-random sampling, and the potential for researcher bias in data interpretation and analysis. These factors make it difficult to replicate the findings.

## Supplementary Information

Additional file 1.

## Data Availability

Upon request, the corresponding author will provide the datasets used and/or analyzed in the current work. However, most of the scripts used in the analysis will be provided as supplementary files.

## Abbreviations

AIDS: Acquired immunodeficiency syndrome
ART: Antiretroviral therapy
C4L-IVR: Call for Life- Interactive Voice Response
FGD: Focus group discussion
HIV: Human Immunodeficiency Virus
IDI: In-depth interview
IMB: Information, Motivation, Behavioral Model
IVR: Interactive voice response
PI: Principal investigator
PMTCT: Prevention of Mother To Child Transmission
Ph.D: Doctor of philosophy
SMS: Short message service
WHO: World Health Organisation
YPLWH: Young people living with HIV

## References

[1] Arayasirikul S, Turner C, Trujillo D, Le V, Wilson EC. Efficacy and impact of digital HIV care navigation in young people living with HIV in San Francisco, California: prospective study. JMIR Mhealth Uhealth. 2020;8(5):e18597. 10.2196/18597. 32383680 10.2196/18597 PMC7244993

[2] Buckingham SA, Walker T, Morrissey K. The feasibility and acceptability of digital technology for health and wellbeing in social housing residents in Cornwall: a qualitative scoping study. Digit Health. 2022;8:20552076221074124. 10.1177/20552076221074124. 35096410 10.1177/20552076221074124 PMC8793427

[3] Bwanika Naggirinya A, Meya DB, Kiragga A, Rujumba J, Waiswa P, Parkes-Ratanshi R. Interactive voice response-call for life mhealth tool effect versus usual care on adherence to anti-retroviral therapy among young people living with HIV: a randomized trial in Uganda. David B. and Kiragga, Agnes and Rujumba, Joseph and Waiswa, Peter and Parkes-Ratanshi, Rosalind, Interactive voice response-call for life mhealth tool effect versus usual care on adherence to anti-retroviral therapy among young people living with HIV: a randomized trial in Uganda. 10.1371/journal.pone.0308923 PMC11575762 39561129

[4] Bwanika Naggirinya A, Meya DB, Nabaggala MS, Banturaki G, Kiragga A, Rujumba J, et al. Effectiveness of interactive voice response-call for life mHealth tool on adherence to anti-retroviral therapy among young people living with HIV: a randomized trial in Uganda. PLoS ONE. 2024;19(11):e0308923. 39561129 10.1371/journal.pone.0308923 PMC11575762

[5] Byonanebye DM, Nabaggala MS, Naggirinya AB, Lamorde M, Oseku E, King R, et al. An interactive voice response software to improve the quality of life of people living with HIV in Uganda: randomized controlled trial. JMIR Mhealth Uhealth. 2021;9(2):e22229. 10.2196/22229. 33570497 10.2196/22229 PMC7906832

[6] Campbell CK, Dubé K, Sauceda JA, Ndukwe S, Saberi P. Antiretroviral therapy experience, satisfaction, and preferences among a diverse sample of young adults living with HIV. AIDS Care. 2022;34(9):1212–8. 10.1080/09540121.2021.2001783. 34793253 10.1080/09540121.2021.2001783 PMC9114167

[7] Endebu T, Deksisa A, Dugasa W, Mulu E, Bogale T. Acceptability and feasibility of short message service to improve ART medication adherence among people living with HIV/AIDS receiving antiretroviral treatment at Adama hospital medical college, Central Ethiopia. BMC Public Health. 2019;19(1):1315. 10.1186/s12889-019-7687-z. 31638936 10.1186/s12889-019-7687-z PMC6805404

[8] Fisher WA, Fisher JD, Harman J. The information-motivation-behavioral skills model: a general social psychological approach to understanding and promoting health behavior. 2003. p. 82–106. 10.1002/9780470753552.ch4

[9] Garofalo R, Kuhns LM, Hotton A, Johnson A, Muldoon A, Rice D. A randomized controlled trial of personalized text message reminders to promote medication adherence among HIV-positive adolescents and young adults. AIDS Behav. 2016;20(5):1049–59. 26362167 10.1007/s10461-015-1192-x PMC4788595

[10] Heylen E, Chandy S, Shamsundar R, Nair S, Ravi Kumar BN, Ekstrand ML. Correlates of and barriers to ART adherence among adherence-challenged people living with HIV in southern India. AIDS Care. 2021;33(4):486–93. 10.1080/09540121.2020.1742862. 32172599 10.1080/09540121.2020.1742862 PMC7492371

[11] Hua D, Petrina N, Young N, Cho JG, Poon SK. Understanding the factors influencing acceptability of AI in medical imaging domains among healthcare professionals: a scoping review. Artif Intell Med. 2024;147:102698. 10.1016/j.artmed.2023.102698. 38184343 10.1016/j.artmed.2023.102698

[12] Kreniske P, Basmajian A, Nakyanjo N, Ddaaki W, Isabirye D, Ssekyewa C, et al. The promise and peril of mobile phones for youth in rural Uganda: multimethod study of implications for health and HIV. J Med Internet Res. 2021;23(2):e17837. 10.2196/17837. 33528375 10.2196/17837 PMC7886611

[13] Masa R, Zimba M, Tamta M, Zimba G, Zulu G. The association of perceived, internalized, and enacted HIV stigma with medication adherence, barriers to adherence, and mental health among young people living with HIV in Zambia. Stigma Health. 2022;7(4):443–53. 10.1037/sah0000404. 36408093 10.1037/sah0000404 PMC9673916

[14] Nalugoda F, Kreniske P, Hofer S, Zhong X, Wei Y, Grilo SA, et al. Cell phones, sexual behaviors and HIV prevalence in Rakai, Uganda: a cross sectional analysis of longitudinal data. AIDS Behav. 2020;24:1574–84. 31520238 10.1007/s10461-019-02665-8 PMC7241097

[15] Saberi P, Lisha NE, Erguera XA, Hudes ES, Johnson MO, Ruel T, et al. A mobile health app (WYZ) for engagement in care and antiretroviral therapy adherence among youth and young adults living with HIV: single-arm pilot intervention study. JMIR Form Res. 2021;5(8):e26861. 10.2196/26861. 34463622 10.2196/26861 PMC8441610

[16] Ssemugabo C, Rutebemberwa E, Kajungu D, Pariyo GW, Hyder AA, Gibson DG. Acceptability and use of interactive voice response mobile phone surveys for noncommunicable disease behavioral risk factor surveillance in rural Uganda: qualitative study. JMIR Form Res. 2019;3(4):e15000. 10.2196/15000. 31793889 10.2196/15000 PMC6918213

[17] St Clair-Sullivan N, Mwamba C, Whetham J, Bolton Moore C, Darking M, Vera J. Barriers to HIV care and adherence for young people living with HIV in Zambia and mHealth. Mhealth. 2019;5:45. 10.21037/mhealth.2019.09.02. 31620472 10.21037/mhealth.2019.09.02 PMC6789205

[18] Tekle A, Tsegaye A, Ketema T. Adherence to anti-retroviral therapy (ART) and its determinants among people living with HIV/AIDS at Bonga, Kaffa, South-West Ethiopia. Patient Prefer Adherence. 2024;18:543–54. 10.2147/ppa.S445164. 38476589 10.2147/PPA.S445164 PMC10929121

[19] Trujillo D, Turner C, Le V, Wilson EC, Arayasirikul S. Digital HIV care navigation for young people living with HIV in San Francisco, California: feasibility and acceptability study. JMIR Mhealth Uhealth. 2020;8(1):e16838. 10.2196/16838. 31922489 10.2196/16838 PMC6996763

[20] Van Rhoon L, McSharry J, Byrne M. Development and testing of a digital health acceptability model to explain the intention to use a digital diabetes prevention programme. Br J Health Psychol. 2022;27(3):716–40. 10.1111/bjhp.12569. 34719099 10.1111/bjhp.12569

[21] Ventuneac A, Kaplan-Lewis E, Buck J, Roy R, Aberg CE, Duah BA, et al. A mobile health intervention in HIV primary care: supporting patients at risk for ART non-adherence. HIV Res Clin Pract. 2020;21(5):140–50. 10.1080/25787489.2020.1862972. 33369547 10.1080/25787489.2020.1862972

[22] Wilbourn B, Howell TH, Castel AD, D’Angelo L, Trexler C, Carr R, et al. Development, refinement, and acceptability of digital gaming to improve HIV testing among adolescents and young adults at risk for HIV. Games Health J. 2020;9(1):53–63. 10.1089/g4h.2018.0162. 31560218 10.1089/g4h.2018.0162 PMC7038573

[23] World Health Organisation. mHealth: use of mobile wireless technologies for public health (139/8). 2016. EB139/8, Issue.

[24] Wutz M, Hermes M, Winter V, Köberlein-Neu J. Factors influencing the acceptability, acceptance, and adoption of conversational agents in health care: integrative review. J Med Internet Res. 2023;25:e46548. 10.2196/46548. 37751279 10.2196/46548 PMC10565637

[25] Yagos WO, Olok GT, Moro EB, Huck J, Nirmalan M. Use of mobile phones for rehabilitative services among prosthetics users in rural Acholi sub-region of northern Uganda: findings from a qualitative study. BMC Med Inform Decis Mak. 2022;22(1):263. 10.1186/s12911-022-02008-z. 36207722 10.1186/s12911-022-02008-z PMC9547425

[26] Zanoni BC, Archary M, Sibaya T, Musinguzi N, Gethers CT, Goldstein M, et al. Acceptability, feasibility and preliminary effectiveness of the mHealth intervention, InTSHA, on retention in care and viral suppression among adolescents with HIV in South Africa: a pilot randomized clinical trial. AIDS Care. 2024;36(7):983–92. 10.1080/09540121.2024.2361240. 38976571 10.1080/09540121.2024.2361240 PMC11269015

