# Supplementary material for: Brief communication: qualitative evaluation of call-for-life mHealth tool among youth living with HIV in Uganda
Source: AIDS Res Ther. 2025 Oct 21;22:107. doi: 10.1186/s12981-025-00798-6 (PMC12542162; doi:10.1186/s12981-025-00798-6)
Supplement: Supplementary file 1 — Additional file 1. [file 12981_2025_798_MOESM1_ESM.pdf]

v. CFL FGD Guide Youth Study

Call for Life Youth Study

Focus Group Discussion Guide

Introduction:

Hi, my name is _____________ and I want to thank you for joining us today. I am helping to coordinate this study on CFLU system here in Kiryandongo. We are conducting this research to identify barriers and enablers of ART adherence among youths in Kiryandongo District and get views about mHealth interventions; what you like, what you don't like and reasons for liking and not liking the system. I would like to say that there are no right or wrong answers in our discussion. We will simply be discussing your views, opinions and experiences on a range of topics, so please feel comfortable to say what you honestly feel. I would like to tape record the whole session. Please do not be concerned about this, all measures will be taken to maintain confidentiality of the interviews and discussions. Information you tell us will ONLY be used for this research project. As we are tape recording the interview, we ask that you refrain from using names or identifying information of yourself or your partners. If at any time during the interview you feel uncomfortable you can ask for a break, refuse to answer any question, and are always free to leave. Do you have any questions before we start?

Create rapport:

How have you been?

<u>mHealth acceptability among youth</u>

Now that you have received information about mHealth /CFLU, tell us what you know about mHealth/CFLU

1. As a youth, would you accept or refuse to use mHealth CFLU tool?
2. Tell us why you would accept or refuse to use mHealth interventions/ CFLU
3. Can you name the specific elements you would like to help in ART adherence? (Probe on pill reminders, health tips, symptom reporting, use of secret pin)
4. How can the system be used? (Probe on timing - during what hours; frequency)
5. What do you ***not*** like about the system? Please describe.
    a. Probe on: reminders, health tips, timing, voice, etc
    b. Probe on why

<u>Barriers, enablers of ART adherence among youths (To phrase questions 4&5 based on ART experience)</u>

1. Tell me everything you know about ART adherence.

2. Who explained to you how to be adherent?
3. Can you think of reasons why youths can't take their medication as they are supposed to do?
4. What is likely to facilitate/ hinder ART adherence/ clinic attendance (ART naives)
5. What facilitated /hindered your adherence to ART/ clinic attendance (ART experienced)

Probe on:

i. Characteristic of individual his/her environment
ii. Treatment regimen
iii. Healthcare providers
iv. Client-provider relationship
v. Health Care setting
vi. Psychosocial issues
vii. Education level
viii. knowledge of HIV ART

Question Guide below 1- 10 to be used only at study end line

1. If you had to rate your comfort level using the system on a Likert scale from 1 to 5 (5 being completely comfortable, and 1 not at all comfortable), how would you rate the experience in general?
2. Can you talk about the reasons you gave the rating? what makes you feel comfortable using and not using the system?
3. How do you think that this system can help patients? Probe on specifics.
4. (if relevant) Can you describe how this system may have helped you take your drugs?
5. Let's talk about the health tips.
    1. How often do you follow the advice of the health tips?
    2. Which ones? Why and why not?
    3. Are there areas you think are missing or you would like more information?
    4. Are there areas you think are not helpful or make you feel uncomfortable?
6. Let's talk about the appointment reminders
    - i. How have they been helpful/not helpful? Please explain.
    - ii. Have you missed an appointment whilst being in the study? Why?
7. Let's talk about the symptom reporting

- How have you used or not used this aspect of the system. If yes, please describe. In your view, what has worked well and what hasn't worked well? What about the waiting? How did you feel while waiting for a call? Is the wait for a call too long or acceptable? Do you like talking to a doctor/nurse? How would you feel about an automated (would need to explain) system?

8. Is there anything you have learned by using the system? Please describe and please be specific.
9. Suggestions: Are there any other things you would like the system to do or not do?
